# Effect of Crevice Size on Crevice Corrosion of N80 Carbon Steel in CO_2_-Saturated NaCl-HAc Solution

**DOI:** 10.3390/ma17164078

**Published:** 2024-08-16

**Authors:** Pengfei Hu, Guangyi Cai, Yizhou Li

**Affiliations:** 1 National Key Laboratory of Science and Technology on Electromagnetic Energy, Naval University of Engineering, Wuhan 430033, China; huhongyi_fei@163.com; 2 East Lake Laboratory, Wuhan 420202, China; 3 Institute of Materials Science and Engineering, Ocean University of China, Qingdao 266100, China

**Keywords:** carbon steel, acidic corrosion, crevice corrosion

## Abstract

The effect of crevice size on the crevice corrosion of N80 carbon steel was investigated by electrochemical measurements and surface analysis in a CO_2_-saturated NaCl-HAc solution. The N80 carbon steel exhibits a high susceptibility to crevice corrosion in this environment, which can be initiated immediately without an induction period for specimens with crevice sizes of 100 μm, 300 μm, and 500 μm. Typically, crevice solutions become more acidic during crevice corrosion; however, in this study, the crevice solution became alkaline, resulting in galvanic corrosion between the inner and outer steel surfaces and leading to severe crevice corrosion. The pH levels of the crevice solution for specimens with 100 μm and 300 μm crevice sizes are similar, but both are notably higher than that of the specimen with a 500 μm crevice size. As a result, there is no significant difference in the crevice corrosion phenomenon between specimens with 100 μm and 300 μm crevice sizes, but it is more severe than in the specimen with a 500 μm crevice size.

## 1. Introduction

Internal corrosion is identified as the primary cause of pipeline failures within the oil and gas industry [1,2,3,4]. The corrosive environment typically consists of water, Cl^−^, CO_2_, H_2_S, and other substances within the pipeline. Acetic acid (HAc) is the most abundant organic acid with a concentration of up to thousands of ppm in the produced aqueous phase. The diverse corrosion types can be induced due to the complicated corrosive species [5,6,7]. Among these, localized corrosion, such as pitting corrosion, crevice corrosion, or stress corrosion cracking (SCC), is considered particularly hazardous [8,9,10]. It has also been reported that most of the corrosion failure of a pipeline is caused by localized corrosion.

Generally, pitting corrosion and crevice corrosion derive from identical corrosion conditions and mechanisms [11,12,13]. However, crevice corrosion is more likely to occur than pitting corrosion due to the shielding effect of crevice structures. The narrow geometry of a crevice can restrict the mass transfer process between the solution inside and outside the crevice, leading to changes in solution chemistry within the crevice. These changes typically involve a decrease in oxygen content and an increase in H^+^ and Cl^−^, which can accelerate the degradation of the passive film and trigger the active dissolution of steel within the crevice [14,15,16]. Therefore, the formation of crevice structures on a steel surface can significantly impact the dynamic corrosion processes of the steel. The development of crevice corrosion is heavily influenced by the geometry of the crevice opening, which dictates the diffusion process between the crevice solution and bulk solution [17,18]. A quick diffusion process can prevent the formation of chemical composition differences (such as oxygen concentration, H^+^, and Cl^−^) between the crevice solutions and bulk solution, then inhibiting the initiation of crevice corrosion. Therefore, a critical crevice opening dimension is necessary for any type of crevice corrosion. Passive metals typically have a small critical crevice opening dimension due to their slow dissolution dynamic process. Decreasing the crevice opening dimension can actually promote crevice corrosion in passive metals. While most studies on crevice corrosion focus on passive metals, there is limited research on active metals [19,20,21]. Previous studies [22,23] have shown that carbon steel is susceptible to crevice corrosion in CO_2_-saturated solutions containing HAc, where the cathodic and anodic reactions of active metals occur more rapidly than those of passive metals. Additionally, there is a lack of oxygen in CO_2_-saturated solutions. This means the crevice corrosion mechanisms and the effect of crevice opening dimensions on crevice corrosion can differ between active and passive metals. However, there is still a lack of research on crevice corrosion in CO_2_-saturated solutions containing HAc and the unclear impact of opening dimensions on crevice corrosion.

In the present study, the effect of crevice size on crevice corrosion in a CO_2_-saturated solution containing HAc was investigated by electrochemical measurements and morphology analysis. The effect of crevice size on the crevice corrosion of N80 carbon steel was discussed. 

## 2. Materials and Methods

### 2.1. Material and Solution

This study utilized N80 carbon steel (Cangzhou Beigang Petroleum Pipeline Co., Ltd., Cangzhou, China) as a typical oil tube material, and its composition (wt.%) is as follows: 0.42% C, 0.24% Si, 1.55% Mn, 0.012% P, 0.004% S, 0.051% Cr, 0.18% Mo, 0.005% Ni, 0.01% Ti, 0.06% Cu, and Fe balance. The yield strength is about 330 MPa and the ultimate tensile strength is about 602 Mpa. Two types of specimens were prepared, one measuring 20 × 10 × 3 mm (an exposed area of 2 cm^2^) for morphology analysis and another measuring 10 × 10 × 3 mm (an exposed area of 1 cm^2^) for electrochemical tests. The specimens were embedded in epoxy resin, abraded with 800-grit silicon carbide paper, degreased with acetone, and cleaned with distilled water.

The test solution consisted of a 1.65% NaCl solution with 600 mg/L of HAc, prepared from analytical grade reagents and deionized water. Prior to testing, the solution was purged with pure CO_2_ gas (99.95%) for 4 h. Specimens were then immersed in the solution with continuous CO_2_ gas-purging at a flux of 20 mL/min to ensure full saturation throughout the test. All experiments were conducted at atmospheric pressure and 30 °C. Each test was repeated at least three times to ensure the reproducibility of the results.

### 2.2. Configuration of the Crevice and Electrochemical Measurements

**Figure 1:**
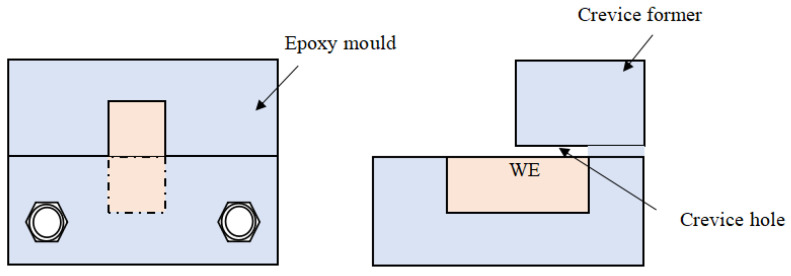
The configuration of the crevice for morphology analysis.

Figure 1 illustrates the schematic diagram of the crevice structure and the setup for morphology analysis. The electrode has an exposed area of 2 cm^2^, with half of it positioned inside the crevice and the other half outside. The size of the crevice was adjusted using PTFE gaskets of varying thicknesses: 100 μm, 300 μm, and 500 μm.

**Figure 2:**
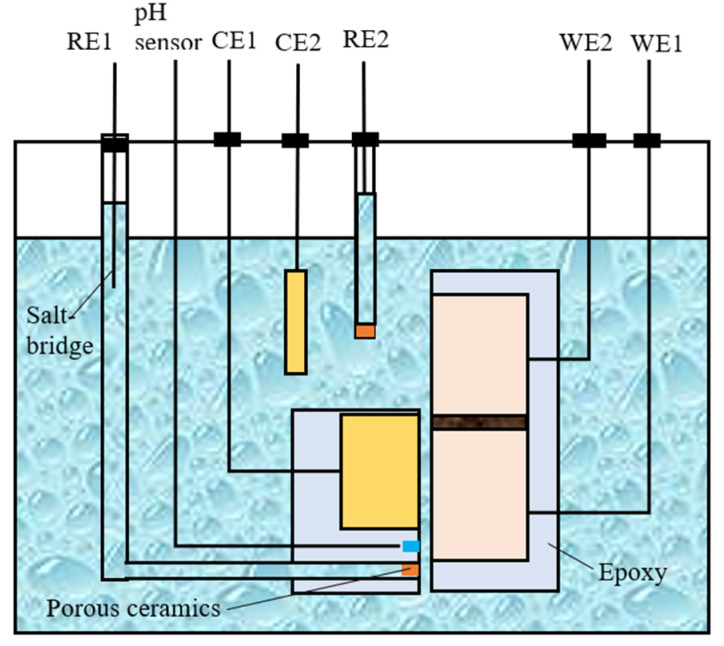
Configuration of crevice and sketch map of setup for electrochemical tests.

The schematic diagram of the setup for open circuit potential and galvanic current measurements is presented in Figure 2. A zero-resistance ammeter (ZRA) was employed to measure the galvanic current, while the OCP measurements were conducted using an electrochemical workstation. 

### 2.3. Morphology Analysis

The optical images of the crevice electrode post-corrosion in the solution were captured using a digital camera. A scanning electron microscope (SEM) (Thermo Fisher Scientific Quanta 200, WLM, Waltham, MA, USA) was employed to analyze the micro corrosion morphology in various regions (inside the crevice, the crevice mouth, and outside the crevice) of the electrode to assess the extent of corrosion. The corrosion groove profiles, post-removal of the corrosion products, were examined using a confocal laser scanning microscope (CLSM, KEYENCEVK-X250, Osaka, Japan).

## 3. Results

**Figure 3:**
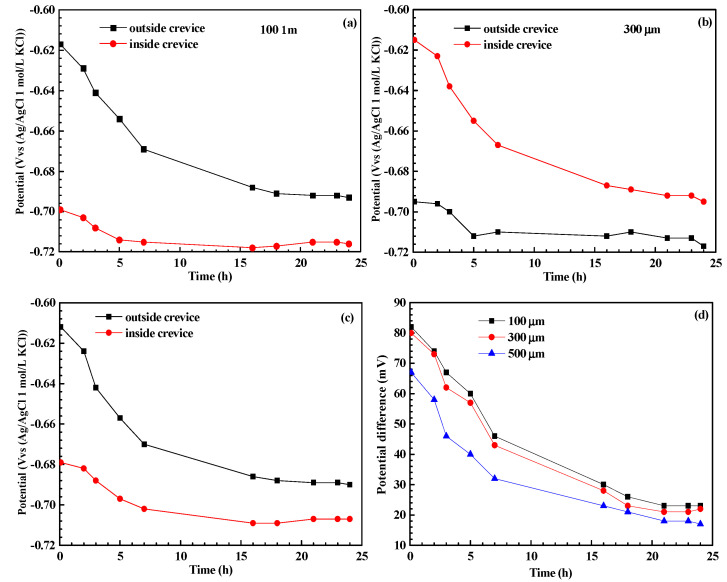
Time dependence of open circuit potential and potential difference between WE1 and WE2 for specimen with different crevice sizes in CO_2_-saturated NaCl–HAc solution: (**a**) 100 μm, (**b**) 300 μm, (**c**) 500 μm, and (**d**) potential difference.

The time dependence of open circuit potential (OCP) and the potential difference between two working electrodes (WE1 and WE2) with varying crevice sizes in a CO_2_-saturated NaCl–HAc solution is illustrated in Figure 3. The OCP evolution of WE1 and WE2 exhibits similar trends across specimens with different crevice opening sizes, gradually decreasing and stabilizing after 24 h of immersion. Additionally, WE2 consistently maintains a more positive OCP compared to WE1 throughout the duration of the experiment, indicating WE2 functions as the cathode, potentially mitigating corrosion, while WE1 serves as the anode, potentially promoting corrosion when connected to each other. Previous studies [22] suggest that the negative OCP within the crevice is attributed to reduced reductive species due to consumption by cathodic reactions and hindered diffusion through the narrow crevice. Moreover, the potential difference between WE1 and WE2 diminishes as crevice size increases, as depicted in Figure 3d, suggesting a gradual increase in galvanic corrosion driving force and the potential promotion of crevice corrosion with smaller crevice openings.

**Figure 4:**
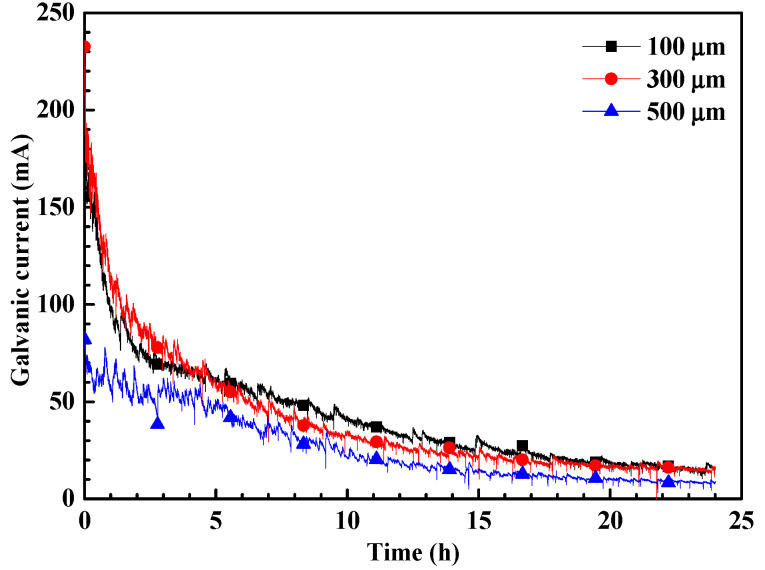
The evolution of the galvanic current of crevice specimens with different crevice sizes in a CO_2_-saturated NaCl–HAc solution.

Figure 4 illustrates the evolution of galvanic current between WE1 and WE2 for specimens with varying crevice opening sizes in a CO_2_-saturated NaCl–HAc solution. The graph demonstrates that the galvanic currents of specimens with different crevice opening sizes initially decrease upon immersion, eventually stabilizing. After 24 h of immersion, all specimens show significant galvanic currents, suggesting that corrosion within the crevice is notably accelerated. The maximum current densities can be observed at the initial immersion, indicating the immediate initiation of crevice corrosion without an induction period. This phenomenon is different from the crevice corrosion of stainless steel in neutral NaCl solution, which usually needs a long induction period to initiate crevice corrosion. Additionally, it is evident that the galvanic currents of specimens with 100 and 300 μm crevice sizes approach each other and are notably higher than those of specimens with a 500 μm crevice size. This suggests that the galvanic corrosion effect between the inner and outer steels is nearly identical for specimens with 100 and 300 μm crevice sizes and is greater than that observed in specimens with a 500 μm crevice size. Therefore, the level of crevice corrosion should be comparable for specimens with 100 and 300 μm crevice sizes, and more severe than that of specimens with a 500 μm crevice size.

**Figure 5:**
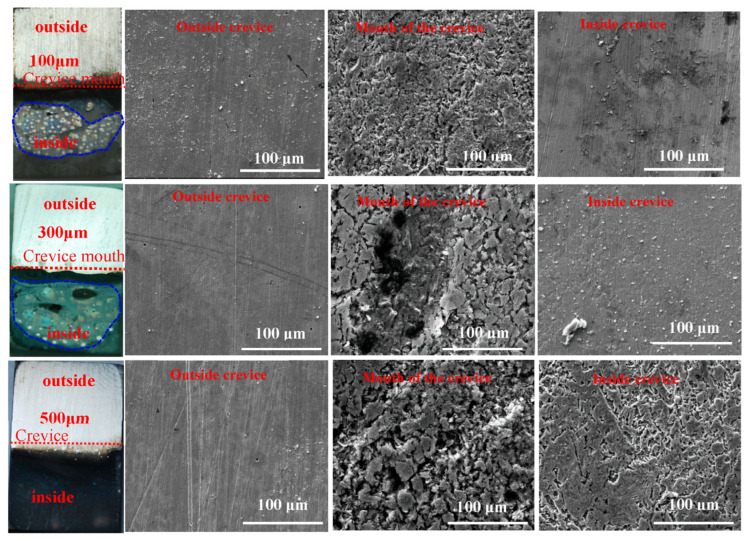
The macro- and micro-morphology of crevice specimens with different crevice sizes in a CO_2_-saturated NaCl–HAc solution.

Figure 5 illustrates the macro-morphology and micro-morphology of specimens with varying crevice opening sizes in a CO_2_-saturated NaCl–HAc solution after 72 h of corrosion. It is observed that the corrosion morphology appears similar across specimens with different crevice opening sizes, with the electrode outside the crevice showing minimal corrosion and retaining a metallic luster. In contrast, the electrode within the crevice experiences severe corrosion, with the most corrosive area located at the crevice mouth. This suggests that crevice corrosion of N80 carbon steel has occurred in specimens with crevice opening dimensions of 100 μm, 300 μm, and 500 μm in a CO_2_-saturated NaCl–HAc solution after corrosion for 72 h.

**Figure 6:**
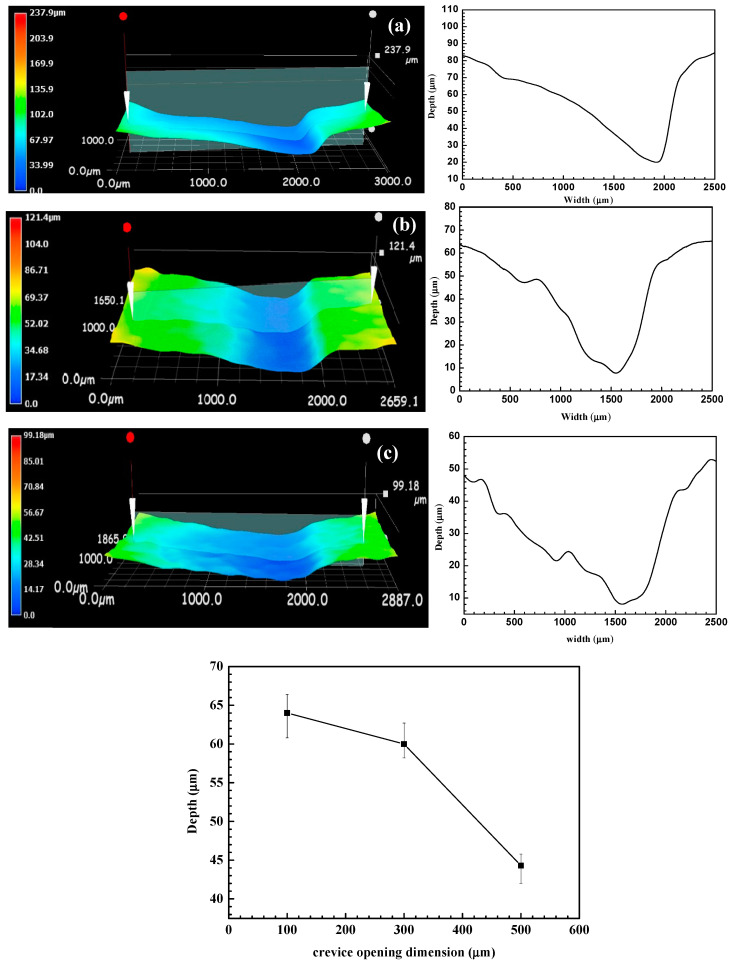
The profile at the crevice mouth of crevice specimens with different crevice sizes in a CO_2_-saturated NaCl–HAc solution,(**a**) 100 μm, (**b**) 300 μm, and (**c**) 500 μm.

The depth of the corrosion groove at the crevice mouth was analyzed using a confocal laser scanning microscope, as depicted in Figure 6. The results show the formation of a distinct localized corrosion groove for specimens with different crevice sizes after 72 h of immersion. The maximum depth of the corrosion groove is about 64 μm, 60 μm, and 44 μm for specimens with 100 μm, 300 μm, and 500 μm crevice sizes, respectively. Obviously, the maximum depth of localized corrosion grooves is close to each other for specimens with 100 μm and 300 μm crevice sizes and is greatly deeper than that for specimens with a 500 μm crevice size. Additionally, the location of the corrosion groove gradually shifts towards the crevice bottom with increasing crevice size, likely due to enhanced diffusion ability with larger crevices. Thus, the location of the maximum concentration gradient will move to the crevice’s bottom direction with the increased crevice size. Typically, crevice corrosion initiates when the crevice opening size is less than 100 μm, but in the case of N80 carbon steel, crevice corrosion can still occur with a 500 μm opening size. This indicates a high susceptibility to crevice corrosion for N80 carbon steel in CO_2_-saturated HAc-NaCl solutions.

**Figure 7:**
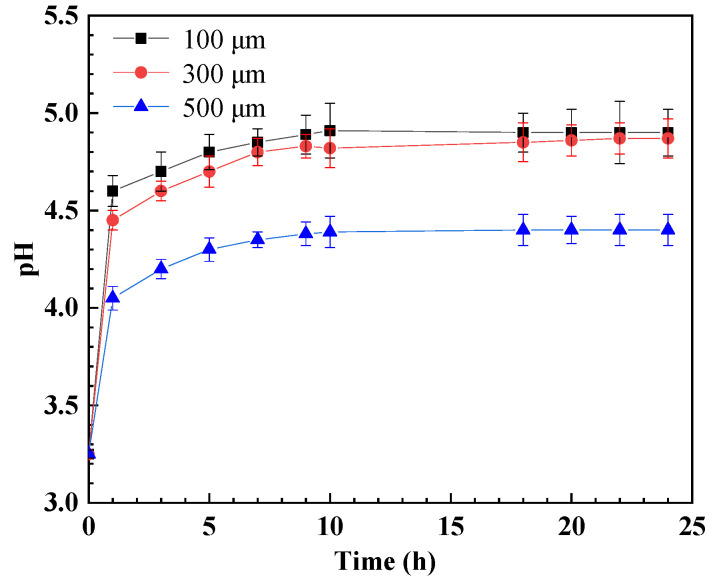
The evolution of pH inside crevices for specimens with different crevice sizes in a CO_2_-saturated NaCl–HAc solution.

Figure 7 illustrates the time-dependent pH values inside and outside a crevice for various crevice opening sizes in a CO_2_-saturated solution containing 600 mg/L of HAc. It is evident that the pH inside the crevice rapidly increases for specimens with different opening sizes following corrosion. This observation aligns with prior research and may be attributed to the consumption of reduction species (H^+^, HAc, H_2_CO_3_, and HCO_3_^−^) by cathodic reactions [24,25]. After 24 h of immersion, the pH inside the crevice for specimens with 100 and 300 μm opening sizes is approximately 4.9, while for the specimen with a 500 μm opening size, it is around 4.4.

## 4. Discussion

### Effect of Crevice Size on Crevice Corrosion of N80 Carbon Steel

It has been reported that in the CO_2_-saturated solution containing HAc, the main cathodic reactions include the following equations [23]:

H^+^ + e- → H (1)

HAc + e- → Ac^−^ + H (2)

H_2_CO_3_ + e- → HCO_3_^−^ + H (3)

HCO_3_^−^ + e- → CO_3_^2−^ + H (4)

The rapid consumption of cathodic reaction species (H^+^, HAc, H_2_CO_3_, and HCO_3_^−^) inside the crevice solution cannot be adequately replenished by diffusion processes. This leads to an elevated pH within the crevice, inhibiting cathodic reactions, as shown in Figure 7. Consequently, the corrosion potential of steel inside the crevice shifts towards a more negative direction, leading to galvanic corrosion between the inner and outer steels, as shown in Figure 3 and Figure 4. The concentration inside the crevice is affected by the consumption rate from cathodic reactions and the diffusion rate between the crevice solution and the bulk solution. Initially, the cathodic reaction rate inside the crevice is comparable for crevices of varying sizes; however, the diffusion rate increases with larger crevice sizes. This indicates that larger crevices require more supplements for cathodic reactants, while the corrosion products can also be transported more quickly from the crevice solution to the bulk solution. This results in a smaller concentration difference between the crevice solution and the bulk solution, as observed in the pH measurements inside the crevice (Figure 7). As a result, larger crevice openings induce a smaller galvanic corrosion driving force and galvanic current, as shown in Figure 3 and Figure 4. Therefore, crevice corrosion is expected to be less severe with larger crevice openings.

However, it should be noticed that the difference in crevice corrosion is not clearly evident between specimens with crevice opening sizes of 100 μm and 300 μm. An examination of Figure 5 reveals that the inner specimen enclosed by the blue dashed line shows minor corrosion, with some bright spots observed in specimens with 100 μm and 300 μm crevice opening sizes. Conversely, specimens within the crevice exhibit severe corrosion in cases where the crevice opening size is 500 μm. This discrepancy can be attributed to the presence of hydrogen bubbles. In this investigation, the primary cathodic reactions are hydrogen evolution reactions, leading to the formation of hydrogen bubbles within the crevice. In specimens with crevice opening sizes less than 300 μm, the hydrogen bubbles struggle to escape the narrow opening and accumulate inside the crevice. The actual crevice opening geometry could be reduced by the hydrogen bubbles inside the crevice, resulting in the small crevice corrosion difference for specimens with 100 μm and 300 μm crevice opening sizes. Conversely, in the case of a crevice opening size of 500 μm, hydrogen bubbles can easily escape through the crevice hole, exerting little influence on the crevice corrosion behavior. Consequently, the crevice corrosion of N80 carbon steel is significantly reduced in specimens with a 500 μm crevice opening size.

## 5. Conclusions

1 The crevice corrosion of N80 carbon steel has a high crevice corrosion susceptibility in a CO_2_-saturated NaCl-HAc solution which could be immediately initiated without an induction period for specimens with 100 μm, 300 μm, and 500 μm crevice sizes.
2 The pH inside the crevice increases rather than decreases after crevice corrosion. This contributes to the negative shift in the corrosion potential of steel inside the crevice, which then triggers the galvanic corrosion effect between the inner and outer metals. Deep corrosion grooves could be formed near the crevice mouth after crevice corrosion.
3 The crevice corrosion phenomenon of N80 carbon steel between specimens with 100 μm and 300 μm crevice sizes has no significant difference, but it is more severe than in the specimen with a 500 μm crevice size.

## Data Availability

The original contributions presented in the study are included in the article, further inquiries can be directed to the corresponding authors.
